# Solubility Enhancement of Budesonide and Statistical Optimization of Coating Variables for Targeted Drug Delivery

**DOI:** 10.1155/2014/262194

**Published:** 2014-04-10

**Authors:** Himanshu Bhatt, Bhargavi Naik, Abhay Dharamsi

**Affiliations:** ^1^National Institute of Pharmaceutical Education and Research (NIPER), B. V. Patel Pharmaceutical Education and Research Development (PERD), S G Highway, Thaltej, Ahmedabad 380054, India; ^2^Department of Pharmaceutics, Maliba Pharmacy College, Uka Tarsadia University, Bardoli-Mahuva Road, Surat District, Gopalvidyanagar 380054, India

## Abstract

The purpose of the research was to present Budesonide (BUD) as a novel formulation showing improved aqueous solubility, which may decrease variability in *C*_max⁡_ and *T*_max⁡_ found in inflammatory bowel disease (IBD) patients, and drug targeting to colon. To improve aqueous solubility, solid dispersion (SD) of the BUD with poloxamer 188 was prepared by melting method. Physical characterization of solid dispersion was performed. The SD was used to prepare tablet equivalent to 9 mg of BUD. The tablet was coated with enteric polymers Eudragit S100 and Eudragit L100 to target colon. The ratio of polymers and percentage coating was optimized using statistical design. Variables studied in design were ratio of enteric polymers and the effect of percentage coating on *in vitro* drug release. Dissolution at different pH showed that drug release in colon could be modified by optimizing the ratio of polymers and percentage coating. The dissolution data showed that the percentage coating and ratio of polymers are very important to get lag time and optimum formulation. The optimized batch from statistical design was kept under accelerated condition for three months. After accelerated stability study, there was no significant change in the drug release.

## 1. Introduction

Inflammatory bowel disease (IBD) involves the inflammation of mucosa in the small and large intestine. In IBD, two conditions persist, for example, Ulcerative colitis (UC) and Crohn's disease (CD). In UC, inflammation occurs into proximal regions of the colon over time. In CD, inflammation occurs to the distal ileum. Thus, there is need of development of drug delivery to both conditions at a time. The formulation design to treat CD will also be beneficial to treat UC. A drug, only in its dissolved form, can be absorbed into stomach and intestine. Drug dissolution occurrs in the distal portions of GIT. In this region, the viscosity of the colonic contents is very high which impede dissolution of poorly water soluble drug [1–3].

Budesonide (BUD) is a potent corticosteroid that is used in the IBD. The absorption variability in the *T*_max⁡_ is very high (30–600 min.) in the patient. Budesonide, BCS Class II, with a log⁡⁡*P* of 3.2, is practically insoluble in water (28 *μ*g/mL) [4] at physiological pH in the intestinal region, which may be the rate limiting for the dissolution and therapeutic potential of budesonide. The objective is to improve the solubility of Budesonide to decrease variability found in *T*_max⁡_. Moreover, bioavailability is only about 20% due to first pass effect. We can minimize variability in *C*_max⁡_ and *T*_max⁡_ by improving the solubility so that the drug concentration can reach faster to their minimum effective level concentration (MEC) for therapeutics efficacy. Solid dispersion technique can improve the oral bioavailability also. Moreover, the intestinal fluid content is less as compared to stomach. So there is need to improve the solubility of BUD. The site of absorption of BUD is also throughout intestinal region. Site specific drug delivery leads to avoidance of first pass metabolism. A statistical design approach was used for optimization of variables which affects drug dissolution. Purpose of the research was to prepare BUD-PXM solid dispersion, as a formulation exhibiting improved aqueous solubility, which may decrease variability in *C*_max⁡_ and *T*_max⁡_ in IBD patients, and to study the combined influence of the independent variables (1) ratio of Eudragit S100 and Eudragit L100 and (2) percentage coating on the dependent variables *Y*_1_ (time required for 50% drug release at pH 6.8), and *Y*_2_ (time required for 100% drug release at pH 7.4).

## 2. Materials and Methods

### 2.1. Materials

The Budesonide (BUD) was a gift sample from Symbiotec Pharma Lab, Indore, India. The Poloxamer 188 (PXM) was obtained from BASF, Ahmedabad, India. Polyvinylpyrrolidone K30 (PVP K30), Croscarmellose sodium (CCS), Sodium Starch Glycolate (SSG), Lactose monohydrate, and Dibutyl phthalate (DBP) were purchased from S. D. Fine Chem. Ltd., Mumbai, India. Eudragit S100 and Eudragit L100 were purchased from Evonik Industries, Mumbai, India. All other reagents and chemicals used in this research were of analytical grade.

### 2.2. Methods

#### 2.2.1. Saturated Solubility Study

The study was carried out by adding excess of drug solution in different solvents, for example, water and phosphate buffer pH 6.8. The saturated solutions were kept on magnetic stirrer for 24 hours at 25°C. After 24 hr, supernatant liquid was taken and filtered through Whatman filter paper (0.45 *μ*). The amount of Budesonide dissolved was quantified by taking supernatant and by making dilution (if required) using UV 1800 (Shimadzu) spectrophotometer at 249 nm.

#### 2.2.2. Preparation of Solid Dispersions

Solid dispersions of BUD were prepared by fusion or melting method [5]. The ratio of BUD-to-polymer (1 : 1) was dispersed in the melted Poloxamer at 55°C. The carrier was heated up to its melting point using controlled water bath. The resultant mixture was immediately cooled to using an ice-water mixture for 2 h. Then mass was allowed to attain at temperature (25–30°C) and stored at room temperature for 24 h. It was pulverized using a glass mortar and pestle and this mass was sifted through a #120 sieve. It was transferred to glass vials and stored at 30°C ± 1°C and the yield was determined using following formula:

(1) $$\begin{array}{c} \text{yield}=\frac{a}{b+c}\times 100, \end{array}$$

where *a* is the weight of the solid dispersion sifted through sieve, *b* is the weight of BUD taken for solid dispersion, and *c* is the weight of various carriers taken for solid dispersion.

#### 2.2.3. Selection of Ratio of BUD-to-PXM

The ratio was selected according to maximum solubilization capacity of drug-to-PXM ratio. The ratio of each of solid dispersions was also characterized by Fourier Transform Infrared Spectroscopy (FTIR).

#### 2.2.4. Preparation of Tablet Core Containing Solid Dispersion

Drug and excipients were mixed geometrically and then granulated using PVP K30 as binder in isopropyl alcohol (IPA). The granules obtained were dried at 50°C for 1 hr in the oven. Dried granules were passed through 22# sieve and the fines were separated using 44# sieve to obtain 22–44# granules. These granules were then lubricated with magnesium stearate (1%) and Talc (2%). The lubricated granules were compressed into tablets using Minipress I tablet compression machine.

### 2.3. Experimental Design

The statistical design was used to study the combined influence of the effect of independent variables like ratio of Eudragit S100, Eudragit L100 (*X*_1_), and percentage coating (*X*_2_) on the dependent variables like time required for 50% drug release at pH 6.8 (*Y*_1_) and time required for 50% drug release at pH 7.4 (*Y*_2_). In this design, 2 factors are studied, each at 3 levels, and experimental runs are performed at all 9 possible combinations [6, 7]. A statistical model incorporating interaction and polynomial terms is used to evaluate the response:

(2) $$\begin{array}{c} Y=b_{0}+b_{1}X_{1}+b_{2}X_{2}+b_{12}X_{1}X_{2}+b_{11}X_{1}^{2}+b_{22}X_{2}^{2}, \end{array}$$

where *Y* is the dependent variable, *b*_0_ is the arithmetic mean response of the nine runs, and *b*_1_ is the coefficient for the factor *X*_1_. The main effect (*X*_1_ and *X*_2_) represents the average result of changing one factor at a time from its low to high value. The interaction terms *X*_1_ *X*_2_ show how the response changes when 2 factors are simultaneously changed. The polynomial terms (*X*_1_^2^ and *X*_2_^2^) are added to find nonlinearity. Two center point batches B10 and B11 are taken in the statistical design.

#### 2.3.1. Preparation of Coating Solution

Coating solution was prepared using different ratios of material like Eudragit L100 and Eudragit S100. Total 15% of polymer concentration was used. Required quantities of polymers were dissolved in mixture of solvents of 5 mL acetone and 5 mL isopropyl alcohol and stirred with magnetic stirrer to get homogeneous coating solution. Dibutyl phthalate was added in above solution as plasticizer (10% on dry polymer based) after getting homogeneous coating solution; coating was done by dipping the tablet in coating solution till desired percentage coating level was achieved and solvent evaporated. The percentage coating was calculated by the following equation:

(3) $$\begin{array}{c} \%\;\text{weight}\;\text{gain}=\frac{W_{t}-W_{o}}{W_{o}}\times 100, \end{array}$$

where *W*_*t*_ is weight of tablet after coating and *W*_*o*_ is initial weight of tablet.

### 2.4. Characterization of Optimized Solid Dispersion

#### 2.4.1. Fourier Transform Infrared Spectroscopy (FTIR)

FTIR spectra of the BUD, PXM, and solid dispersion were recorded using a Fourier Transform Infrared spectrophotometer (FTIR-ATR system by Bruker Alpha with OPUS-software). Samples were prepared using KBr (spectroscopic grade) disks by means of hydraulic press. The samples were scanned from 4000 to 500 cm^−1^.

#### 2.4.2. Thermal Analysis

DSC spectra of drug and its carrier were recorded in DSC Shimadzu 60 with TDA trend line software. Sample of each of 10 mg was accurately weighed using Sartorius MC5 electronic microbalance, sealed in aluminium DSC pans, and placed in the DSC chamber. Thermal traces were obtained by heating from 50°C to 300°C at heating rate of 10°C per minute under nitrogen atmosphere (100 mL/min) in empty crucibles. An empty aluminium pan was used as reference.

#### 2.4.3. X-Ray Powder Diffraction (XRPD) Studies

About 100 mg of sample was sprinkled over glass slide containing grease to make a layer having thickness of ~0.5 mm. The study was performed by an X-ray diffractometer (PANalytical, XPERT-PRO, New Zealand). The sample slide was put vertically at zero angle degree in the sample chamber. An X-ray beam (Cu target X-ray tube) of 2 kV was allowed to fall over the sample. As the slide moves at an angle of theta degree, a proportional detector detects diffracted X-rays at angle of 2-theta degrees. XRPD patterns were recorded using XPERT-PRO software.

#### 2.4.4. Drug Content in Solid Dispersion

The drug content of solid dispersion was determined by dissolving 40 mg weighed dispersion in 100 mL phosphate buffer pH 6.8 followed by agitation with a magnetic stirrer for 15 minutes to extract the drug. After filtration through Whatman filter paper (0.45 *μ*), the drug concentration in the phosphate buffer pH 6.8 was determined using UV 1800 (Shimadzu) spectrophotometer at 249 nm for Budesonide [8]:

(4) $$\begin{array}{c} \%\;\text{Drug}\;\text{content}=\frac{\text{Calculated}\;\text{drug}\;\text{concentration}}{\text{theoretical}\;\text{drug}\;\text{concentration}}\times 100. \end{array}$$

### 2.5. Evaluation of Granules

#### 2.5.1. Micromeritics Properties of Granules [9]

Various micromeritic parameters like angle of repose, bulk density, tap density, Carr's (Compressibility) Index (CI), and Hausner's ratio were measured.

### 2.6. Evaluation of Core Tablet

#### 2.6.1. Appearance, Size, Shape, Thickness, and Diameter of Tablet

10 tablets were taken and their thickness and diameter were measured by using Vernier Callipers and average of the diameter and thickness was calculated.

#### 2.6.2. Weight Variation Test [10]

20 tablets were weighed separately using Digital electronic balance and the test was performed according to the official method in Indian Pharmacopeia 2010.

#### 2.6.3. Hardness Test [11]

Hardness indicates the ability of a tablet to withstand mechanical shocks while handling of it. Hardness of core tablets was determined using validated Pfizer hardness tester. It is expressed in kg/cm^2^. Three tablets were randomly picked from batch and analyzed for hardness. The mean and standard deviation were also calculated.

#### 2.6.4. Friability Test [12]

The tablet equivalent to 6.5 g was taken and this test was performed using the Roche friabilator. Friability can be determined by following equation:

(5) % friability   =Initial  wt.  of  tablets−wt.  of  tablets  after  testInitial  weight  of  tablets×100.

#### 2.6.5. Content Uniformity of Core Tablets [13]

10 tablets were individually assayed. Each tablet was crushed individually and added into 100 mL volumetric flask containing 5 mL methanol and 25 mL phosphate buffer pH 6.8. It was sonicated for 5 min and volume was made up to 100 mL with phosphate buffer pH 6.8. The solution was filtered through 0.45 *μ*m Whatman filter paper. The filtrate was suitably diluted with phosphate buffer pH 6.8 and analyzed by UV 1800 (Shimadzu) spectrophotometer at 249 nm [8].

### 2.7. *In Vitro* Dissolution Studies of Factorial Batches

Coated tablets containing equivalent to 9 mg of BUD were used for the dissolution studies. The study was performed using USP I basket apparatus at 37°C ± 0.5°C at 75 rpm. The dissolution media was 900 mL of 0.1 N HCl for 2 hours, acetate buffer pH 4.6 for 2 hours, phosphate buffer pH 6.8 for 3 hours, and pH 7.4 until complete drug release (*n* = 3). A 5 mL amount of dissolution media was withdrawn at intervals of 1, 2, 3, 4, 5, 6, 7, 8, 9, and 10 hours. An equal amount of fresh dissolution media was replaced immediately after withdrawal of the test sample. Test samples were filtered through a 0.45 *μ*m membrane filter (Sartorius, Hamburg, Germany) and suitably diluted. The absorbance of each diluted sample was measured at 249 nm using a double beam UV-1800 spectrophotometer (Shimadzu, Japan).

### 2.8. Kinetic Study and Mechanism of Drug Release [14, 15]

To get the release mechanism of drug from coated tablets, release study data were subjected to statistical analysis by zero-order, first-order, Higuchi, and Korsmeyer Peppas equations.

### 2.9. Accelerated Stability Study

The optimized batches of coated tablets were kept under accelerated condition of 40°C ± 2°C/75% ± 5% RH for three months in Osworld JRIC-11B stability chamber. Tablets were evaluated periodically (0, 1, 2, and 3 months) for appearance, content uniformity, and* in vitro *drug release.

## 3. Results and Discussion

### 3.1. Saturated Solubility Study

The solubility of pure BUD in distilled water and phosphate buffer pH 6.8 was found to be 0.085 ± 0.0003 and 0.0429 ± 0.0078, respectively. It indicates that the drug is practically insoluble. Therefore, a solid dispersion technique using PXM was employed for dissolution enhancement of BUD. The results for solubility study in different solvents are reported in Table 3.

### 3.2. Preparation of Solid Dispersion

After the preparation, different ratios of drug-to-polymer like 1 : 1, 1 : 2, 1 : 3, 1 : 4, and 1 : 5, the percentage yield, and drug content were calculated. The percentage drug content was found to be maximum in drug-to-polymer ratio of 1 : 3 which complies with the assay limit. The results of percentage yield and drug content are shown in solid dispersion as in Table 4.

### 3.3. Selection of Ratio of Drug-to-Polymer

**Figure 1:**
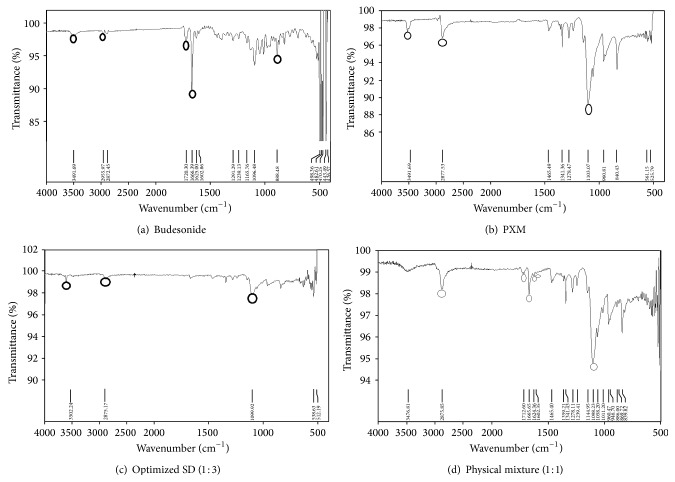
IR spectra of (a) BUD, (b) PXB, (c) optimized SD, and (d) Physical mixture.

BUD may exist in the solid dispersion in 2 different forms, namely, crystalline and amorphous. The dissolution rate of solid dispersion depends on the proportion of the 2 forms, which in turn depends on the proportion of PXM in the solid dispersion. An enhancement of dissolution of BUD because of the proportion of the amorphous form of BUD may increase because of increase in weight fraction of PXM up to its saturated solubility [16, 17]. The solubility study was done in water in different ratios of BUD and PXM. The ratio 1 : 3 showed maximum solubility as compared to other ratios. The solubility decreased beyond 1 : 3 due to increase in proportion of the crystalline form of BUD in solid dispersion at higher ratio. The data of saturated solubility of solid dispersion are shown in Table 4 and FTIR of optimized solid dispersion as shown in Figure 1(c).

### 3.4. Characterization of Optimized Solid Dispersion

#### 3.4.1. Fourier Transform Infrared Spectroscopy

The FTIR spectrum of BUD, PXM, and solid dispersion is shown in Figures 1(a), 1(b), and 1(c), respectively. The characteristic peaks of pure BUD at 3491.69, 2955.97, 1720.30, 1666.39, and 888.48 cm^−1^ are assigned due to stretching of O–H, C–H, C=O, C=C, and C–H (aromatic ring) groups. The PXM exhibits characteristic peaks at 3503, 2884, and 1114 cm^−1^ due to stretching of O–H, C–H, and C–O groups. The peak at 3491, 1720m and 1666 cm^−1^ of the O–H, C=O, and C=C is the important characteristics of PUB. The characteristic stretching bands of pure drug and PXM were shifted at 2878, 1712, and 1097 cm^−1^ in FTIR spectra of optimized batch SD3. Shifting of the peak intensity clearly indicates the interaction of drug with carriers due to strong or weak H-bond formation which improves dissolution. In physical mixture (Figure 1(d)) of BUD and PXM, all the parent peaks of BUD and PXM appear.

#### 3.4.2. Differential Scanning Calorimetry (DSC)

**Figure 2:**
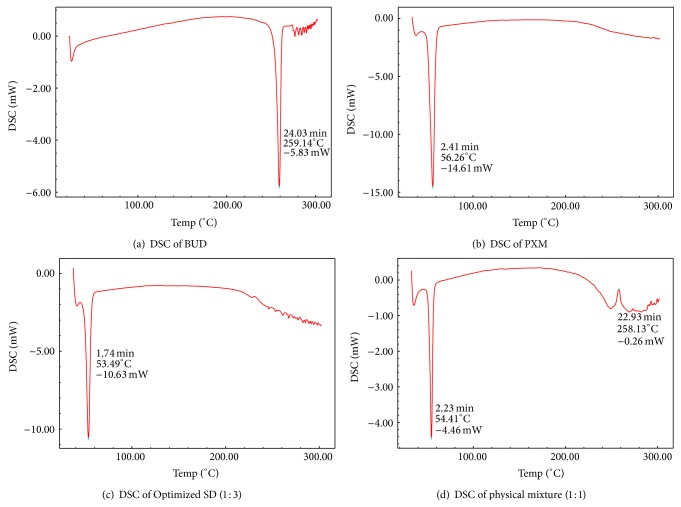
DSC thermogram of (a) BUD, (b) PXM, (c) optimized SD, and (d) physical mixture.

The DSC of Budesonide (Figure 2(a)) and poloxamer 188 (Figure 2(b)) showed sharp endothermic peak at 259.14°C and 56.26°C which corresponds to melting point of drug and polymer. The DSC of the physical mixture (Figure 2(d)) showed two peaks, indicating the melting points of carrier (Poloxamer 188) at 54.41°C and drug, respectively. In physical mixture, there was no change in peak of drug at 259.14°C, which reveals that there is no interaction between drug and carrier. The intensity of melting peak of drug was reduced in physical mixture due to dilution effect. DSC thermogram of solid dispersion (Figure 2(c)) of BUD with poloxamer 188 in ratio 1 : 3 showed single endothermic peak at 53.49°C which is the melting endothermic peak of Poloxamer 188 indicating that there is some interaction between polymer and drug which is necessary for the solid dispersion. No melting peak of drug at 259°C appeared in this thermogram indicating the complete dispersion of the drug in the carrier polymer due to phase transition.

#### 3.4.3. X-Ray Powder Diffractometry (XRPD)

**Figure 3:**
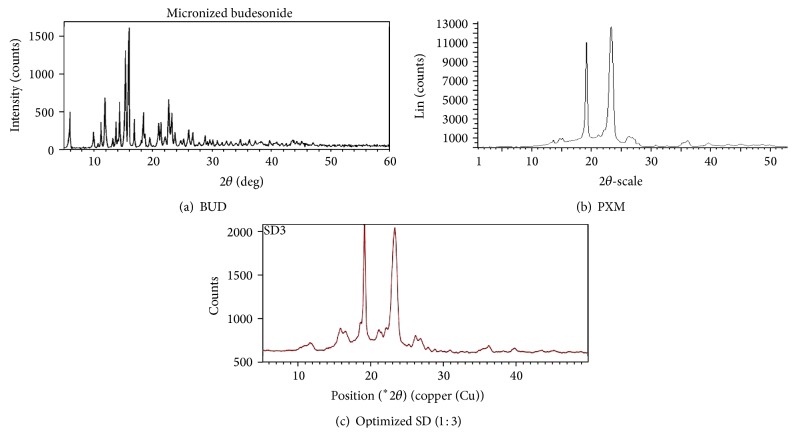
X-Ray diffractograms of (a) BUD, (b) PXM, and (c) optimized SD (1 : 3).

The X-ray diffractogram of pure Budesonide (Figure 3(a)) clearly showed the peak indicating that the drug is in crystalline form. The peak intensity of drug in solid dispersion (Figure 3(c)) was reduced, indicating that the drug was converted into amorphous nature. In the X-ray diffractograms of BUD, sharp peaks at a diffraction angle of 5°, 10°, 11°, 12°, 15°, 16°, and 22° indicate the crystallinity of the drug. The solid dispersion showed sharp peaks at 11.7°, 16°, and 16.5° revealed that some of the crystalline peaks of the drug were still detectable but with reduced intensity and less number in the diffractogram. This data confirms that the little amount of crystalline drug is still present in the solid dispersion although the complete disappearance of its melting peak in the corresponding DSC curves. The sharp drug peaks of drug are absent in the diffractogram of solid dispersion. This indicates that crystallinity of drug is reduced in the solid dispersion which leads enhancement of dissolution of the drug.

### 3.5. Evaluation of Granules

Angle of repose less than 35° indicates good flow property and value of angle of repose for the prepared batch was found in limit which indicates good flow property of granules. Compressibility index which was found to be 14.81% indicates good compressibility. Hausner's ratio which was found to be 1.17 indicates good compressibility. The results of micromeritic properties of granules are shown in Table 5.

### 3.6. Evaluation of Core Tablet

The hardness values of formulations were within the range of 3-4 kg/cm^2^. Friability values of all the formulations were less than 1%. In determination of tablet weight variation, less than 7.5% weight variation is acceptable in the tablet formulation having average weight less than 250 mg. All the formulations were found to be within pharmacopoeial limits as per weight variation test. The content uniformity in all core tablets was found to be within limit of 94.44–113.33% which complies with pharmacopoeial limit of 85–115%. The results are shown in Table 6.

### 3.7. Optimization of Coating Variables by 3^2^ Full Factorial Design

The translation of coded values of variables is in actual units in Table 1. The coating compositions of factorial batches (B1 to B11) are shown in Table 2.

### 3.8. *In Vitro* Dissolution Study of Factorial Batches

**Figure 4:**
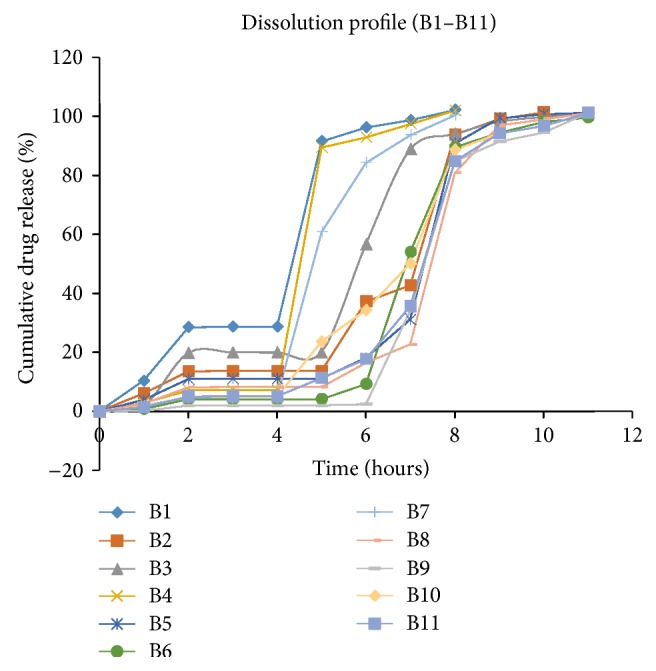
*In vitro* release study of factorial batches.

The factorial batches were prepared by using independent variables like ratio of Eudragit S100 and Eudragit L100 (*X*_1_) and % weight gain (coating) (*X*_2_) and to check its effect on dependent variables like *Y*_1_ and *Y*_2_. Factorial batches of Budesonide were evaluated for the* in vitro* drug release and by its regression analysis. The cumulative percentage of Budesonide release rate for all the formulations (B1 to B11) are shown in Figure 4.

The dissolution data revealed that, as the ratio of Eudragit S100 : Eudragit L100 increases, drug release at 7 hour and 10 hour decreases, and as the percentage coating increases, the cumulative drug release decreases. Collectively as both the factors, ratio of Eudragit S100 : L100, and % coating level increase, it increases the lag time. Eudragit S100 is being soluble around pH 7.0 which leads to the formation of pores in the coating layer which allows medium to diffuse into the core tablet and ruptures the outer coat. Thus, the level of coating and the concentration of enteric polymer play a very important role for optimizing the formulation.

### 3.9. Statistical Analysis

#### 3.9.1. Fitting Data to the Model

A two-factor, three-level factorial statistical design was employed as the response surface methodology requires 11 experiments. All the responses observed for 11 formulations prepared were fit to quadratic model using Design Expert software 8.0.7.1. The best fit model was quadratic model and the comparative values of *R* ^2^, adjusted *R* ^2^, predicted *R* ^2^, *P* lack of fit (LOF), adequate precision, S.D., and %CV are given in Table 7 along with the regression equation generated for each response in Table 9. A positive value represents an effect that favors the optimization, while a negative value indicates an inverse relationship between the factor and the response. It is evident that both independent variables, namely, the concentration of Eudragit S100 : Eudragit L100 (*X*_1_) and % weight gain (*X*_2_) have significant effects on the three responses, namely, drug release at 7 hr (*Y*_1_) and 10 hrs (*Y*_2_).

Data given in Tables 7 and 8 demonstrates that all the models were significant at 5% confidence level since *P* values were less than 0.05. The lack of fit (LOF) *F*-test describes the variation of the data around the fitted model. If the model does not fit the data well, this will be significant. The large *P* values for lack of fit (>0.05) *P*-lack of fit (LOF) indicated that the *F*-statistic was insignificant, which implies significant model correlation between the variables and responses. For all the models, the predicted *R* ^2^ value was in reasonable agreement with the adjusted *R* ^2^. Adequate precision (AP) compares the range of the predicted values at the design points to the average prediction error. For all response, ratio greater than 4 indicates adequate model discrimination and all predicted models can be used to navigate the design space. The coefficient of variance (CV) is the ratio of the standard error of estimate to the mean value of the observed response defines reproducibility of the model. Here, CV value was found to be less than 10% and it confirms that all models are reproducible.

#### 3.9.2. Data Analysis of *Y*_1_ (%CDR at 7 Hour)

The observed value for %CDR at 7 hour for all 11 batches B1–B11 varied from 22.69% to 98.67%. The result clearly indicates that *Y*_1_ is strongly affected by the independent variables selected for the study. The response (*Y*_1_) obtained at various levels of two independent variables were subjected to multiple regression to give a quadratic polynomial equation:

(6) Y1=32.32−18.80X1−13.30X2−12.41X1X2+44.44X12+1.47X22.

As the factor *X*_1_ increases, the response *Y*_1_ decreases and this is indicated by negative coefficient value of dependent variable. These two variables *X*_1_ (*P* < 0.05) and *X*_2_ (*P* < 0.05) were found to be significant in affecting *Y*_1_. The negative coefficient value for independent variable *X*_1_ (−18.80) indicates the negative effect on the dependent variable *Y*_1_, for example, increase in Eudragit S100 : Eudragit L100 ratio will lead to decrease in %CDR at 7 hour. The negative coefficient value for *X*_2_ (−13.30) indicates the negative effect on %CDR at 7 hour, for example, increase in Eudragit S100 : Eudragit L100 leads to decrease in %CDR at 7 hour.

#### 3.9.3. Data Analysis of *Y*_2_ (%CDR at 10 Hour)

The observed value for %CDR at 10 hour for all 11 batches B1–B11 varied from 94.44% to 101.26%. The result clearly indicates that *Y*_2_ is strongly affected by the independent variables selected for the study. The response (*Y*_2_) obtained at various levels of two independent variables was subjected to multiple regression to give a quadratic polynomial equation:

(7) Y2=100.49−2.02X1−1.57X2−0.86X1X2−0.67X12−0.57X22.

As the factor *X*_2_ increases, the response *Y*_2_ decreases and it was indicated by negative coefficient value of dependent variable. These two variables *X*_1_ (*P* < 0.05) and *X*_2_ (*P* < 0.05) were found to be significant in affecting *Y*_2_. The negative coefficient value for independent variable *X*_1_ (−2.02) indicates the negative effect on the dependent variable *Y*_1_, for example, increase in percentage coating will lead to decrease in %CDR at 10 hours. The negative coefficient value for *X*_2_ (−1.57) indicates the negative effect on %CDR on 10 hours; that is, increase in percentage coating leads to decrease in %CDR at 10 hour.

### 3.10. Contour Plots and Response Surface Analysis

**Figure 5:**
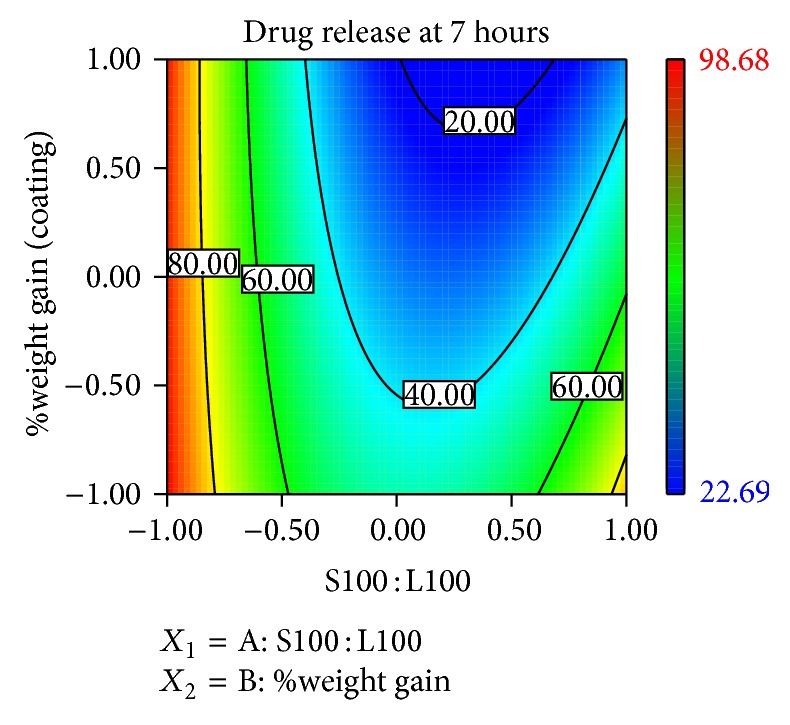
2D contour plot of *Y*_1_.

**Figure 6:**
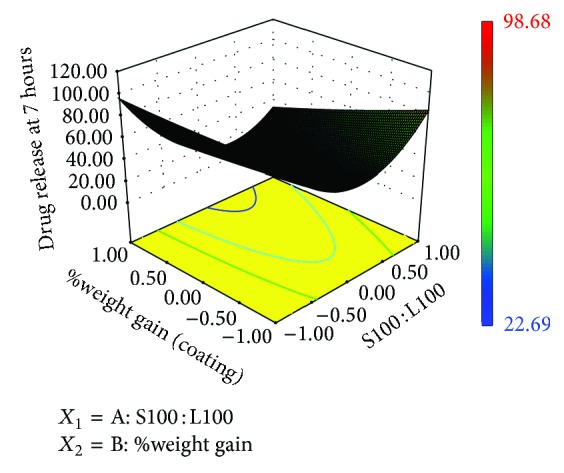
Response surface plot of *Y*_1_.

**Figure 7:**
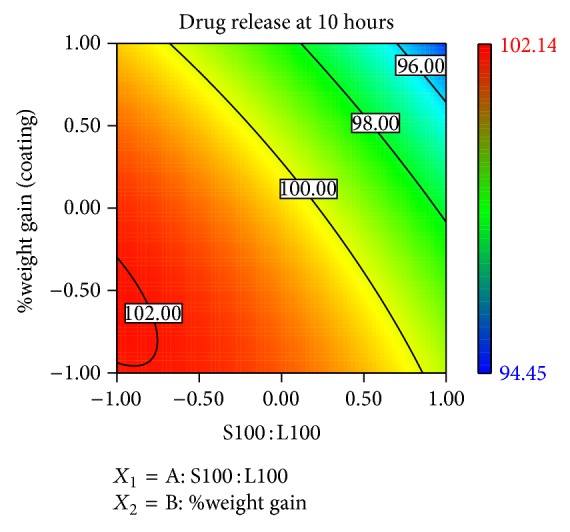
2D contour plot of *Y*_2_.

**Figure 8:**
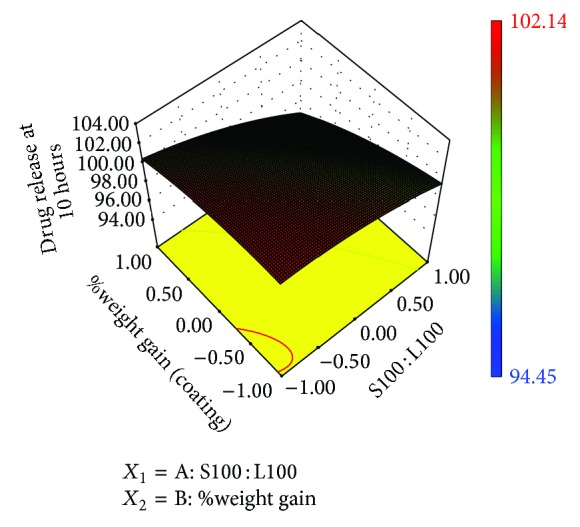
Response surface plot of *Y*_2_.

**Figure 9:**
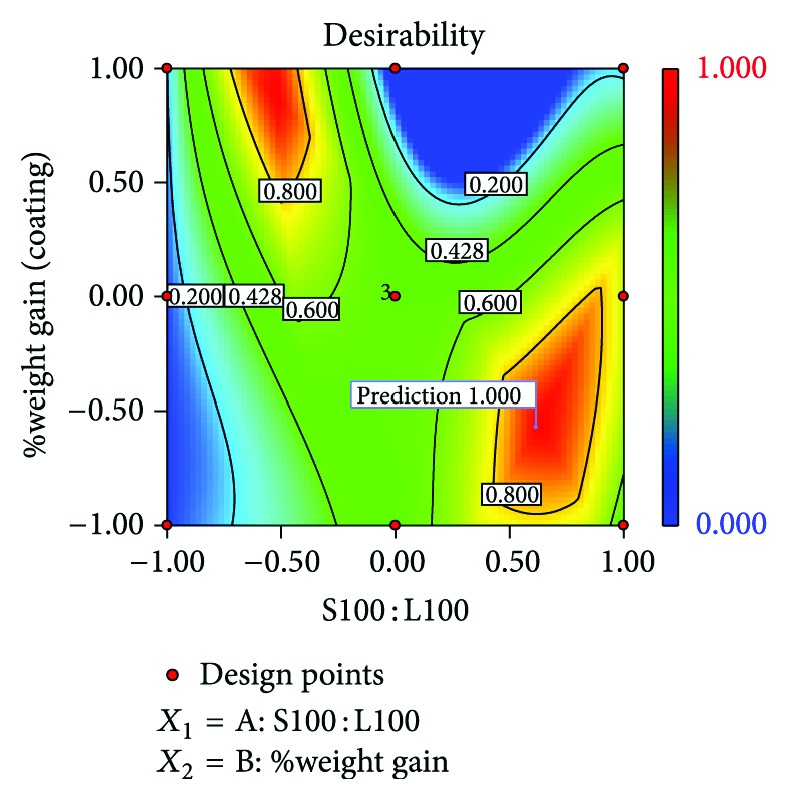
Desirability plot of optimized batch HB1.

**Figure 10:**
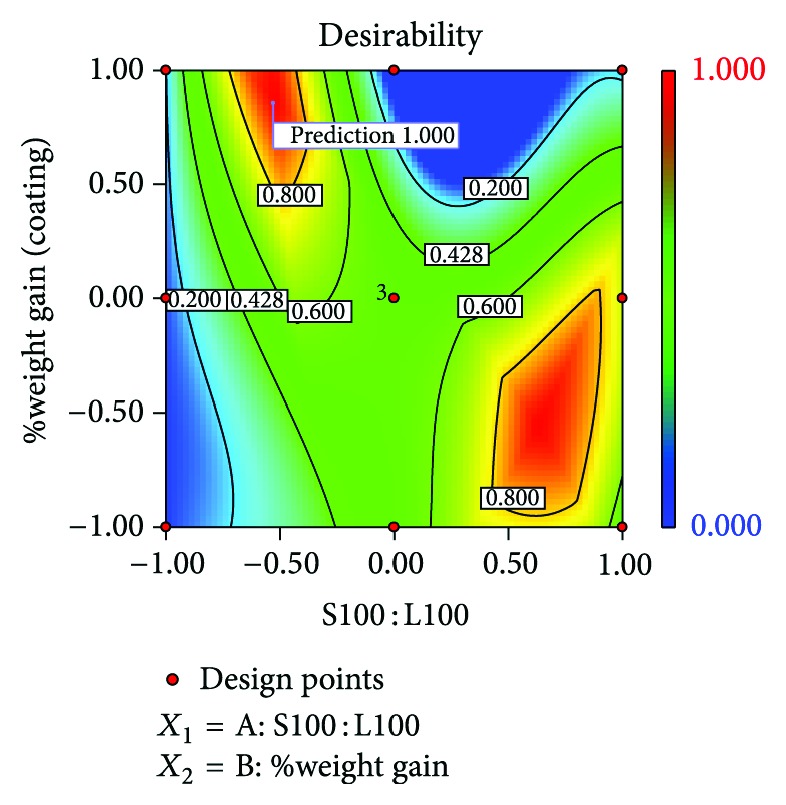
Desirability plot of optimized batch HB2.

**Figure 11:**
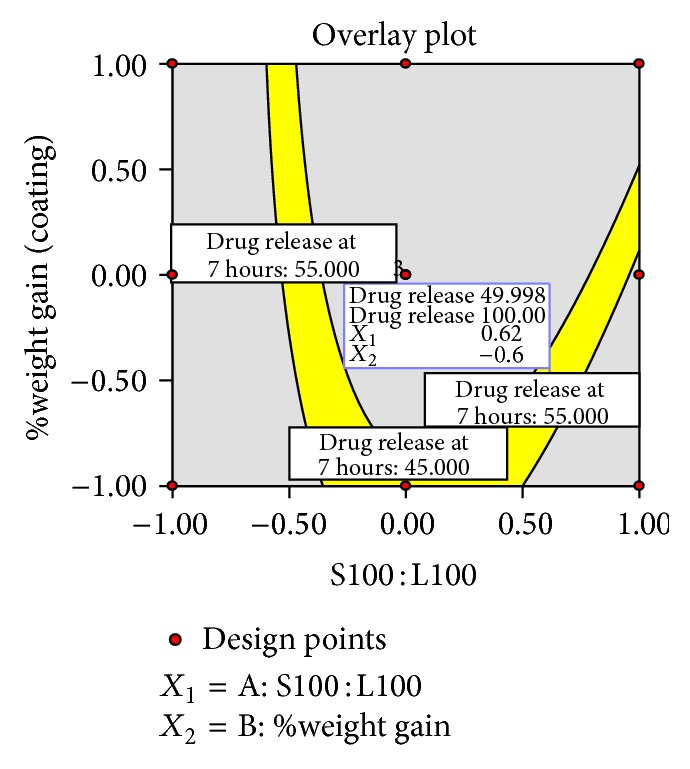
Overlay plot of optimized batch HB1.

**Figure 12:**
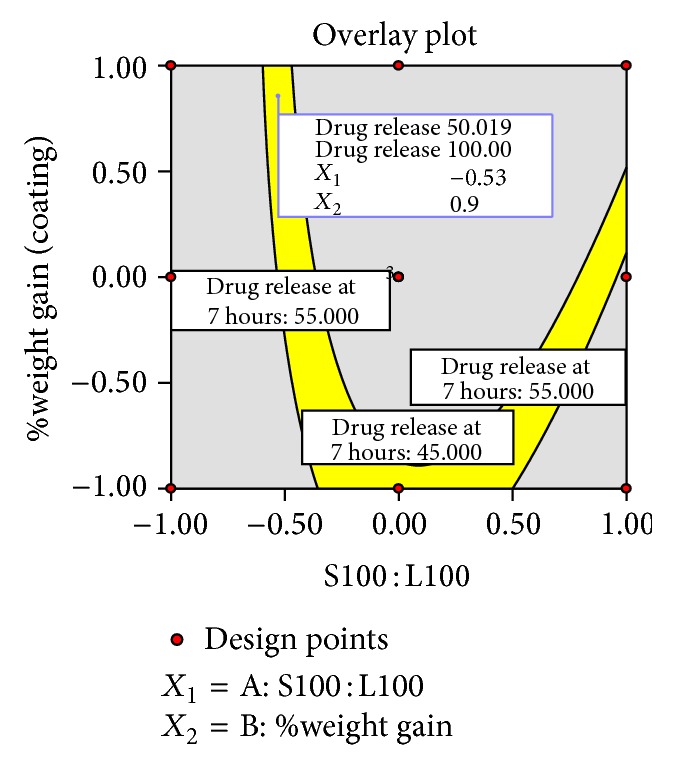
Overlay plot of optimized batch HB2.

Two-dimensional contour plots and 3D response surface plots are shown in Figures 5, 6, 7, 8, 9, 10, 11, and 12 which are very useful to study the interaction effects of the factors on the responses. These types of plots are useful in the study of the effects of two factors on the response at one time. All the relationships among the two variables are nonlinear, although they exhibit a nearly linear relationship as shown in Figures 5, 6, 7, 8, 9, 10, 11, and 12.

### 3.11. Optimization

The optimum formulation was selected based on the criteria of attaining the constraints of variables response as shown in Table 11. Upon trading of various response variables and comprehensive evaluation of feasibility search and exhaustive grid search, batch was considered as an optimum batch HB1 which was composed of coating polymer ratio of Eudragit S100 : Eudragit L100 (81 : 19) and 6.05% weight gain (coating). Another optimum batch HB2 was found to be in the ratio of Eudragit S100 : Eudragit L100 (23.5 : 76.5) and 9.5% weight gain (coating). The compositions of optimized batches are shown in Table 10.

### 3.12. *In Vitro* Dissolution Study of Optimized Batches

**Figure 13:**
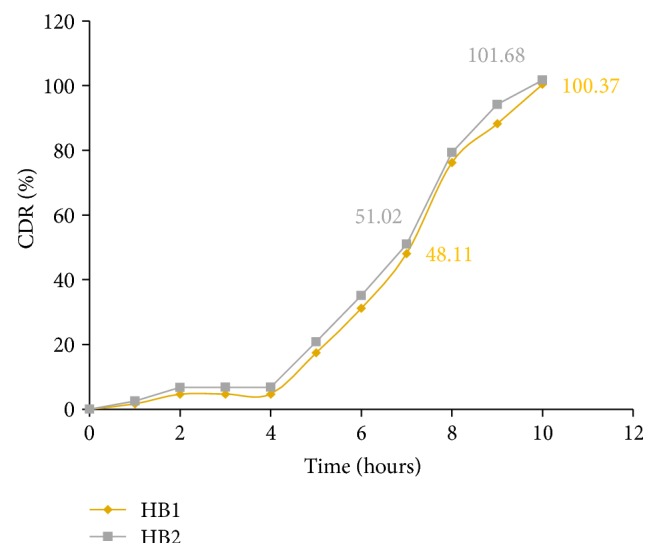
*In vitro* dissolution profile of optimized batch HB1 and HB2.

For each batch of the coated tablets, three tablets were subjected to the dissolution studies.* In vitro* dissolution studies were performed for the prepared tablet formulations. The data were shown in Figure 13.

### 3.13. Kinetic Modeling and Mechanism of Drug Release of Optimized Batches

These different kinetic equations were applied to interpret the release rate from all the formulations. The best with higher correlation coefficient (*R* ^2^ = 0.9489) and (*R* ^2^ = 0.9503) was found with first-order drug release.

### 3.14. Validation of Response Surface Methodology

Polynomial models including interactions and quadratic terms were generated for all the response variables of coating using multiple linear regression analysis. In order to assess the reliability of the developed mathematical model, formulations corresponding to random compositions of experimental domain were performed. For each of these formulations, the responses were estimated by the use of generated mathematical models and by the experimental procedures.

To validate the chosen experimental design and polynomial equations, two additional random batches of coating composition and percentage coating were formulated in experimental matrix to determine the validity of the model generated. These validation batches are also called as check-point batches. Subsequently, the result and experimental values of various responses were compared quantitatively with predicted values of responses. The % error (%PE) in prognosis was calculated using the formula:

(8) $$\begin{array}{c} \%\text{PE}=\frac{\text{Experimental}\;\text{value}-\text{Predicted}\;\text{value}}{\text{Experimental}\;\text{value}}*100. \end{array}$$

The compositions of batches covering the entire range of experimental domain are depicted in Table 12. Tablet enlists the composition of the check-point batches, their predicted values and experimental values of all the response variables, and %PE in prognosis for sustained release. For both the checkpoint batches, the results of dependent variables (*Y*_1_ − *Y*_2_) were found to be within limits. Table 12 showed the composition of checkpoint formulations, their predicted and experimental values for all response variables, and %PE in prognosis. The %PE was calculated as it is helpful in establishing the validity of generated equations and to describe the domain of applicability of RSM model. For validation of experimental design results, the experimental values of the responses were compared with the anticipated values. The prediction error was found to vary between −0.022 and 0.069. Hence, these results demonstrate the reliability of the optimization procedure in predicting the effect of process variables.

### 3.15. Accelerated Stability Study of Optimized Batch

Tablets were evaluated periodically (0, 1, 2, and 3 months) for appearance, drug content, and* in vitro *drug release. No significant changes were observed in any of the study parameter during study period, indicating stability of HB1 and HB2 batch. Results of stability study are given in Tables 13, 14, and 15.

## 4. Conclusion

The present investigation deals with the colon targeting of binary solid dispersion of Budesonide and optimization of ratio for coating using Eudragit S100 and Eudragit L100 and % weight gain. The Poloxamer 188 was used as carrier to improve the solubility of Budesonide which may reduce patient to patient absorption variability in patients of Ulcerative Colitis and Crohn's disease. Combination of Eudragit S100 and Eudragit L100 was used for enteric coating and to target the drug to ileum and colon. Optimization of coating variables was done using factorial design at 3 levels and 2 factors. From the polynomial equation and contour plots generated, the two independent factors showed significant effect on dependent variables. The drug release was delayed until the formulation reach around at pH 6.8 and above which is physiological pH of ileum and colon. It was good fit to the first-order kinetic. The optimized batches HB1 and HB2 exhibited the selected dissolution criteria. Thus, the improvement in solubility of Budesonide is achieved using Poloxamer 188 and colon targeting is achieved using Eudragit S100 and Eudragit L100. It is suitable to get site-specific delivery and delay release.

## Figures and Tables

**Table 1:** Translation of coded values of variables in actual units.

Translation of coded values in actual units			
Independent variables	Variable level		
	Low (−1)	Medium (0)	High (+1)
Eudragit S100 : Eudragit L100	0 : 100	50 : 50	100 : 0
% weight gain (coating)	5%	7.5%	10%

Dependent variables			
*Y*_1_ = % CDR at 7 hours			
*Y*_2_ = % CDR at 10 hours			

**Table 2:** Coating composition of factorial batches (B1 to B11).

Ingredients	Eudragit S100 (g)	Eudragit L100 (g)	Dibutyl phthalate (mL) (10% of total polymer weight)	Isopropyl alcohol (mL)	Acetone (mL)	% weight gain (coating)
B1	—	3	0.3	10	10	5
B2	1.5	1.5	0.3	10	10	5
B3	3	—	0.3	10	10	5
B4	—	3	0.3	10	10	7.5
B5	1.5	1.5	0.3	10	10	7.5
B6	3	—	0.3	10	10	7.5
B7	—	3	0.3	10	10	10
B8	1.5	1.5	0.3	10	10	10
B9	3	—	0.3	10	10	10
B10∗	1.5	1.5	0.3	10	10	7.5
B11∗	1.5	1.5	0.3	10	10	7.5

^*^Center point batches.

**Table 3:** Solubility data in different solvents.

Solvent	Solubility (mg/mL) (mean ± S.D.)	Solubility (mg/mL)
Distilled water	0.0385 ± 0.0003	Practically insoluble
Phosphate buffer pH 6.8	0.0429 ± 0.0078	Practically insoluble

**Table 4:** % yield, drug content and solubility of different ratio of drug-to-polymer.

Batch	Ratio of drug-to-polymer	% yield	% drug content	Solubility (mg/mL) (mean ± S.D.)
SD1	1 : 1	96.32 ± 1.78	97.44 ± 0.113	0.7573 ± 0.0515
SD2	1 : 2	97.23 ± 0.78	93.95 ± 1.109	0.8593 ± 0.0131
**SD3**	**1 : 3**	94.73 ± 1.52	98.24 ± 1.015	0.9462 ± 0.1980
SD4	1 : 4	93.78 ± 0.42	96.88 ± 0.674	0.7753 ± 0.0071
SD5	1 : 5	96.91 ± 1.65	98.03 ± 0.443	0.4882 ± 0.1180

**Table 5:** Precompression study of granules.

Angle of repose (mean ± S.D.)	Bulk density (mean ± S.D.)	Tap density (mean ± S.D.)	Carr's Index (CI)	Hausner ratio
33.98 ± 1.42	0.69 ± 0.027	0.81 ± 0.037	14.81	1.17

**Table 6:** Postcompression parameters of core tablet.

Parameters	Results (mean ± S.D.)
Average weight	200.8 ± 0.27
Content uniformity	100.74 ± 0.79
Deviation	(94.44% to 113.33%)
Friability	0.729 ± 0.081
Hardness	3.66 ± 0.15
Thickness	3.15 ± 0.040

**Table 7:** Summary of results of regression analysis for responses *Y*_1_ and *Y*_2_ for fitting to quadratic model.

Statistical parameters	*Y*	
	*Y*_1_	*Y*_2_
*P* value	<0.0001	0.0003
*P* LOF	0.1481	0.2669
*R* ^2^	0.9921	0.9804
Adjusted *R* ^2^	0.9842	0.9608
Predicted *R* ^2^	0.9267	0.8420
Adequate precision	27.308	22.938
Standard deviation	3.84	0.42
% C.V.	6.69	0.42
PRESS	686.03	7.24

**Table 8:** Summary of results of multiple regression analysis for response *Y*_1_ and *Y*_2_.

Dependent variable	*Y*_1_ (% release at 7 hour)		*Y*_2_ (% release at 10 hour)	
	*P* value	Coefficient	*P* value	Coefficient
Intercept	—	32.32	—	100.49
*X*_1_	<0.0001	−18.80	<0.0001	−2.02
*X*_2_	0.0004	−13.30	0.0003	−1.57
*X*_12_	0.0013	−12.41	0.0096	−0.86
*X*_1_^2^	<0.0001	44.44	0.0539	−0.67
*X*_2_^2^	0.5679	1.47	0.0852	−0.57

**Table 9:** Summary of Quadratic polynomial equation for responses *Y*_1_ and *Y*_2_ for fitting to quadratic model.

*Y*	Mathematical model
*Y*_1_	*Y*_1_ = 32.32 − 18.80*X*_1_ − 13.30*X*_2_ − 12.41*X*_1_ *X*_2_ + 44.44*X*_1_^2^ + 1.47*X*_2_^2^
*Y*_2_	*Y*_2_ = 100.49 − 2.02*X*_1_ − 1.57*X*_2_ − 0.86*X*_1_ *X*_2_ − 0.67*X*_1_^2^ − 0.57*X*_2_^2^

**Table 10:** Coating composition of optimized batches.

Ingredients	Quantities	
	Batch HB1	Batch HB2
Eudragit S100	2.43 g	0.705 g
Eudragit L100	0.57 g	2.295 g
Isopropyl alcohol (mL)	10 mL	10 mL
Acetone (mL)	10 mL	10 mL
Dibutyl phthalate (mL) (10% of total polymer weight)	0.3	0.3
% weight gain (coating)	6.05%	9.5%

Total	20 mL	20 mL

**Table 11:** Results of optimized batch HB1 and HB2 for response variables.

Batch	Response variables	Constrains	Predicted value	Experimental value
HB1	*Y*_1_ = % CDR at 7 hours	45 ≤ *Y*_1_ ≥ 55	50%	48.11%
	*Y*_2_ = % CDR at 10 hours	95 ≤ *Y*_2_ ≥ 100	100%	100.36%

HB2	*Y*_1_ = % CDR at 7 hours	45 ≤ *Y*_1_ ≥ 55	50%	51.01%
	*Y*_2_ = % CDR at 10 hours	95 ≤ *Y*_2_ ≥ 100	100%	101.67%

**Table 12:** Composition of checkpoint formulations, the predicted and experimental values of response variables, and % prediction error.

Checkpoint batch composition (A : B)	Response variable	Experimental value	Predicted value	% PE
(−0.5 : 0.5)	*Y*_1_ = % CDR at 7 hours	48.55	49.66	−0.022
	*Y*_2_ = % CDR at 10 hours	100.18	100.62	−0.004

(0.5 : −0.5)	*Y*_1_ = % CDR at 7 hours	47.45	44.15	0.069
	*Y*_2_ = % CDR at 10 hours	100.78	100.17	0.006

**Table 13:** Results of stability study of optimized batch HB1 and HB2.

Tested after time	HB1		HB2	
	Appearance	Drug content (%) (Mean ± S.D.)	Appearance	Drug content (%) (mean ± S.D.)
0 month	No change	100.18% ± 2.10	No change	98.7% ± 1.39
1 month	No change	99.09% ± 0.63	No change	98.97% ± 0.74
2 months	No change	98.72% ± 1.30	No change	99.34% ± 1.12
3 months	No change	98.12% ± 0.98	No change	99.87% ± 1.89

**Table 14:** *In vitro* drug release study of HB1 after stability.

Time (hours)	% Drug release (mean ± S.D.)			
	0 month	1 month	2 month	3 month
1	1.64 ± 0.59	2.01 ± 0.20	1.12 ± 0.78	2.91 ± 0.89
2	4.64 ± 1.30	3.94 ± 0.25	3.98 ± 0.33	4.98 ± 0.78
3	4.68 ± 1.30	3.94 ± 0.25	3.98 ± 0.33	4.98 ± 0.78
4	4.68 ± 1.30	3.94 ± 0.25	3.98 ± 0.33	4.98 ± 0.78
5	17.40 ± 1.66	17.72 ± 0.43	18.78 ± 1.21	19.12 ± 1.45
6	31.16 ± 0.51	33.81 ± 1.22	33.67 ± 1.89	32.49 ± 1.56
7	48.11 ± 0.65	48.54 ± 0.89	49.09 ± 0.63	49.94 ± 0.98
8	76.24 ± 0.80	75.81 ± 0.98	78.11 ± 1.12	79.42 ± 0.92
9	88.19 ± 2.79	88.47 ± 2.18	90.41 ± 1.97	91.75 ± 2.22
10	100.36 ± 0.87	100.98 ± 0.76	101.72 ± 1.12	101.12 ± 1.59

**Table 15:** *In vitro* drug release study of HB2 after stability.

Time (hours)	% Drug release (mean ± S.D.)			
	0 month	1 month	2 months	3 months
1	2.54 ± 0.79	2.93 ± 0.83	3.14 ± 0.45	3.45 ± 1.09
2	6.75 ± 1.49	6.09 ± 0.30	5.92 ± 0.91	6.12 ± 1.21
3	6.80 ± 1.49	6.09 ± 0.30	5.92 ± 0.91	6.12 ± 1.21
4	6.80 ± 1.49	6.09 ± 0.30	5.92 ± 0.91	6.12 ± 1.21
5	20.82 ± 2.03	21.18 ± 0.24	22.41 ± 1.78	22.89 ± 1.98
6	35.14 ± 2.50	35.83 ± 0.27	37.77 ± 2.10	36.39 ± 1.91
7	51.01 ± 0.95	51.83 ± 0.30	52.49 ± 0.56	50.42 ± 0.71
8	79.33 ± 0.72	80.61 ± 0.18	81.66 ± 1.11	82.48 ± 1.39
9	94.15 ± 0.40	94.65 ± 1.08	93.90 ± 0.90	92.60 ± 0.97
10	101.67 ± 1.97	102.35 ± 0.42	102.71 ± 2.22	102.11 ± 2.56
